# Work-Related Self-Efficacy and Illness Identity in Adults with Autism

**DOI:** 10.3390/ijerph20010122

**Published:** 2022-12-22

**Authors:** Liron Lamash, Sonya Meyer

**Affiliations:** 1 Department of Occupational Therapy, University of Haifa, Haifa 3498838, Israel; 2 Department of Occupational Therapy, Ariel University, Ariel 4077603, Israel

**Keywords:** autism spectrum disorder, identity, work-related self-esteem, quality of life

## Abstract

Finding and retaining employment significantly challenges individuals with autism spectrum disorder (ASD). The employment rates of individuals with ASD are described as extremely low, barred by various environmental, occupational, and personal factors. Illness identity is how a person’s health condition integrates with their identity and daily life and relates to self-esteem, employment, and quality of life. Adults with ASD may experience challenges developing positive identities within social and work environments, but illness identity has not been studied among this population. This study examines the autism identity of adults with ASD and the relationships to their self-reported work-related self-efficacy and quality of life. Seventeen participants aged 19–47 years diagnosed with ASD completed the Illness Identity Questionnaire, Work-Related Self-Efficacy Scale and World Health Organization Quality of Life Assessment. Participants reported significantly higher autism acceptance feelings. Negative feelings about living with autism were significantly correlated to lower work-related self-efficacy. Higher levels of enrichment feelings were significantly associated with a higher quality of life. These findings highlight the effect of illness identity on the work-related self-efficacy and quality of life among individuals with ASD. Allied health professionals and educators can assist these individuals in raising their awareness of how they perceive their autism, and in promoting its positive perception.

## 1. Introduction

Autism spectrum disorder (ASD) is a neurodevelopmental disorder characterised by impairments in social and communication skills and restricted and repetitive behaviour or interests [1]. These characteristics typically continue into adulthood, and most adults with ASD have challenges participating in their daily lives and achieving independence [2]. Work is an important occupation in adulthood, and employability is a major essential skill needed in adulthood and a significant factor in the individual’s identity and quality of life [3,4]. The literature indicated that employment is highly meaningful for individuals with ASD [5,6]. However, individuals with autism face significant challenges in finding and retaining employment. The employment rates of individuals with ASD are described as extremely low and barred by various environmental, occupational and personal factors [7,8,9].

Although very little is known about effective ways to enhance adults with ASD in obtaining and maintaining jobs [10], occupational therapists play a crucial role in supporting individual and environmental outcomes to promote this population’s employment [11]. There are relationships between the employability experience of individuals with ASD and their abilities to advocate for their diagnosis and needs and request adjustments in the workplace [12,13]. Moreover, self-advocacy and asking for needed workplace accommodations can help improve job participation and productivity while supporting self-efficacy perception and welfare [14,15]. The literature further showed that individuals with ASD have challenges disclosing their condition and requesting workplace adjustments [16]. However, it provided less information about how individuals with ASD perceive their autism.

Self-efficacy refers to a person’s belief in their ability to organise and execute the regulated actions and behaviours required to perform [17]. However, few studies examined subjective self-perceptions among adults on the autism spectrum [18]. Most importantly, the very little research examining work-related self-efficacy among employees with ASD indicated they have lower work-related self-efficacy than do neurotypical adults [12,19].

Identity is a complex definition of the self that includes an interpersonal aspect, possibilities or potential and values. Self-identity is often created by society and shaped by the individual due to social negotiation [20]. Identity formation is a dynamic, multidimensional, lifelong process that influences the individual’s life decisions. A cohesive identity enables people to engage in purposeful, regulated, autonomous behaviour. It allows them to recognise their strengths and limitations and believe in their ability to control their lives and function as successful adults in society [21]. In addition, self-identity serves as a guide to everyday behaviours and choices, such as career choices, and closely relates to self-efficacy and psychological well-being [22,23].

Illness identity is the degree to which a person’s health or developmental condition is integrated into their identity and daily life [24]. Illness identity also relates to how much the disease or diagnosis is integrated into the individual’s social relationships [25,26]. Oris et al. [24] used that term to describe the level at which chronic illness fits into a person’s self-esteem. Those authors sought to understand why some people with chronic illness experience difficulty coping with daily living activities, whereas others more easily cope with the same challenges.

People with accompanying health conditions or neurodevelopmental diagnoses need to understand the meaning of their health condition relative to their identity to create or regain a sense of self-coherence related to how they deal with the difficulties around them [27,28]. Illness identity has been studied widely among individuals with chronic illness [24,29,30] but not yet among individuals with ASD.

Adults with ASD may have challenges developing positive identities within the social environment, which may not understand or accept them. The medical model perspective, which influenced scientific and educational research, refers to autism as a pathology needing a cure [31]. On the other hand, autism acceptance movements and organisations based on the disability studies perspective refer to autism as contributing to society’s diversity [32,33].

The aim of this study is to examine (1) the initial internal reliability of the Illness Identity Questionnaire (IIQ) among adults with ASD and (2) the relationship between components of their autism identity and their self-reported work-related self-efficacy and quality of life.

## 2. Materials and Methods

### 2.1. Participants and Procedure

We conducted a cross-sectional study using computerised questionnaires in collaboration with ESHNAV Ltd., an organisation that provides various services for special needs populations to support employment and career development. ESHNAV trains and mentors clients to integrate them into the general work force according to their personal abilities, not limited to any specific fields or professions. All ESHNAV Ltd. clients participating in this program had a diagnosis of ASD according to DSM-5 and currently in a training program or already integrated in the work world and mentored by ESHNAV. The level of ASD severity was not provided. Nevertheless, all the participants in this study had the ability to read, write and complete the online questionnaires independently. Suitable participants were sent an invitation via the ESHNAV Ltd. mailing list to participate in the study. Volunteering participants signed a written informed consent form and completed all questionnaires for this study. Ultimately, the sample included 17 adults aged 18 years or older, diagnosed with ASD and able to complete the questionnaires independently. The ESHNAV Ltd. organisation explained the study and invited participation. ESHNAV referred volunteers with ASD who agreed to participate in the study to sign an online informed consent form and provided a link to an online survey created using Qualtrics. Participation was voluntary and completely anonymous. We collected data from April 2021 to June 2021. The study was approved University of Haifa, Faculty of Social Welfare & Health Sciences ethics committee (approval number 274/19, 25 July 2019).

### 2.2. Measures

#### 2.2.1. Demographic Questionnaire

Participants provided basic demographic information, including gender, age, living environment, education level, employment and income level, on the demographic questionnaire.

#### 2.2.2. Illness Identity Questionnaire 

The IIQ consists of 25 items [24]. It was developed based on the theoretical foundations of the four illness identity components it assesses: rejection (five items), engulfment (eight items), acceptance (five items) and enrichment (seven items). Participants indicate the degree of agreement with each IIQ question on a 5-point Likert scale (i.e., 1 = strongly disagree to 5 = strongly agree). The mean score for each of the four IIQ components are calculated. The rejection and engulfment components depict less illness integration, while the acceptance and enrichment components depict more adaptive illness integration. A higher IIQ total mean score, represents a more positive sense of illness identity. The IIQ was initially validated in young people with type 1 diabetes [24], then among additional young adults and adults diagnosed with various chronic health conditions [29,34,35]. High factorial discriminate validity was confirmed along with good reliability (α = 0.75 to α = 0.95) for each of the four factors in the diverse populations. The Hebrew version of the IIQ used in this study had been validated previously [30]. In accordance with the IIQ authors’ guidelines [24] we replaced the term illness with the term autism for the purpose of this study (e.g., Item 7: ‘My illness is part of who I am’). The respondents’ illness identity profile was produced by calculating a normalised category score (percent) to attain a graphical presentation of the IIQ profile components. Mean scores for negative (rejection and engulfment) and positive (acceptance and enrichment) components were also calculated. Lastly, a total mean score of all the IIQ questions (after reverse coding negatively worded questions) to achieve a continuous scale of the IIQ. 

#### 2.2.3. Work-Related Self-Efficacy Scale

The Work-Related Self-Efficacy Scale (WSS) is a validated 37-item self-report scale that measures self-efficacy in four relevant activity domains: vocational service access and career planning, job acquisition, work-related social skills and general work skills [36]. For each item, participants circle a confidence rating on a scale of 0% to 100%. The WSS yields a total score on a 100-percentage-point scale, with higher scores indicating stronger efficacy. The WSS-37 has strong construct reliability and concurrent validity, and the internal consistency is sufficient for each factor (α = 0.85–0.94).

#### 2.2.4. World Health Organization Quality of Life Assessment

The World Health Organization Quality of Life Assessment (WHOQOL-BREF) is an abbreviated questionnaire for assessing subjective quality of life. This questionnaire measures four areas related to quality of life: physical health, psychological health, social relationships, and environment. It also provides a general assessment of a person’s quality of life and health. The questionnaire has good reliability and validity and is used worldwide [37].

### 2.3. Data Analyses

We analysed the data with IBM SPSS Statistics (ver. 26.0). Descriptive statistics were calculated to describe ranges, means, and standard deviations of the participants’ demographic data and of the measures. Following a one-sample Kolmogorov–Smirnov test, normal distribution was revealed in all dependent variables. Therefore, we conducted paired-sample *t* tests to evaluate the differences between components. Cohen’s *d* was calculated for effect size (0.10 indicates a small effect, 0.30 indicates a medium effect and 0.50 indicates a large effect) [38]. We used Pearson correlations to evaluate correlations among illness identity (IIQ), work-related self-efficacy (WSS) and quality of life (WHOQOL-BREF). 

## 3. Results

### 3.1. Participant Characteristics

Seventeen adults aged 19 through 47 years (M = 28.29 years, SD = 8.9) who had been diagnosed with ASD participated in this study. Thirteen (76.5%) were men, and four (23.5%) were women, similar to the male-to-female ratio in the prevalence of ASD worldwide [39].

Most (n = 9) participants lived with their parents or family members, six reported living in community housing and residential support programs, and two reported living independently. Fifteen participants reported being single, and two reported being in a relationship. All participants were proficient in the Hebrew language. 

### 3.2. Illness Identity Characteristics

**Figure 1:**
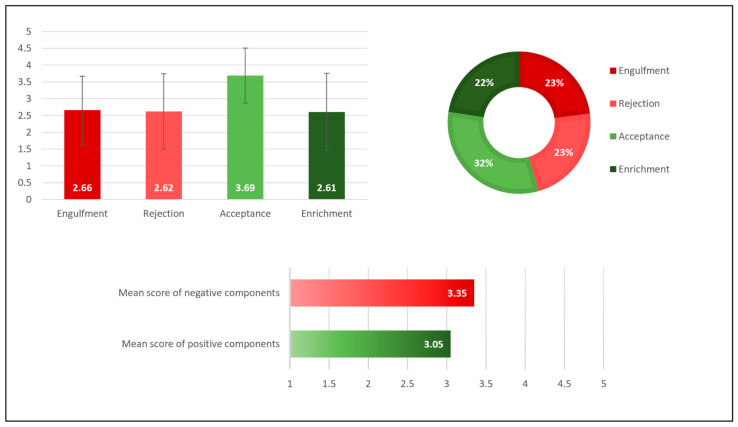
Illness Identity Components and Profile of the Adults with Autism Spectrum Disorder.

The internal reliability of all the IIQ questions ASD version was 0.91, and moderate to high for each of the four components (0.67–0.89). The IIQ total mean score was 3.21 (SD = 0.77). Table 1 presents the remaining descriptive statistics of the four IIQ components. We created the illness identity profile by calculating the components’ relative fraction of the total score (see Figure 1).

No significant difference was found between the positive IIQ mean scores and the negative IIQ mean scores, *t*(16) = 1.32, p = NS, *d* = 0.32, suggesting the participants reported similar positive feelings about their ASD (*M* = 3.058, *SD* = 0.89) as their negative feelings (*M* = 3.35, *SD* = 0.92). Though, paired-sample t tests showed that the feeling of acceptance was significantly higher than the feeling of rejection, *t*(16) = −2.45, *p* < 0.05, *d* = 0.57, the engulfment component, *t*(16) = 2.79, *p* < 0.05, *d* = 0.65, and the enrichment component, *t*(16) = 4.23, *p* < 0.01, *d* = 1.00. No significant differences were found among the other illness identity components.

### 3.3. Correlation between IIQ and Age 

Pearson correlations were calculated to analyze correlations between the IIQ components and the participants’ age, age at diagnosis and years since diagnosis. No significant correlation was found between IIQ components or total score and age, age at diagnosis or time of exposure to diagnosis.

### 3.4. Correlation between Illness Identity and Work-Related Self-Efficacy

Cronbach’s alphas for all WSS-37 items in the current study were 0.96 and high to excellent for the four domains (0.82–0.92). Participants rated the WSS-37 items on a scale of 0 to 100%, higher WSS-37 total mean score represents a more positive work-related self-efficacy. The total mean score of the WSS-37 was 66.72 (*SD* = 18.73). Table 2 presents the remaining descriptive statistics of the four WSS-37 domains.

Significant correlations were observed between the IIQ total score and the WSS-37 total score (*r* = 0.55, *p* < 0.05). As the participants sensed a more positive illness identity, they had higher work-related self-efficacy. Continued tests revealed that engulfment was significantly correlated with the work-related social skills domain (*r* = −0.65, *p* < 0.01). Hence, the more participants sensed engulfment feelings following their autism diagnosis, the lower their reported self-efficacy in social skills required for work. We found no significant correlations between engulfment and the other WSS-37 domains. In addition, enrichment was significantly correlated with the work-related social-skills domain (*r* = 0.50, *p* < 0.05). The more participants sensed enrichment feelings following their autism, the higher they reported self-efficacy of their social skills required for work. No significant correlations were found between enrichment and the other WSS-37 domains or between the IIQ rejection and acceptance components and the WSS-37 domains.

### 3.5. Correlations between IIQ Components and the WHOQOL-BREF

Cronbach’s alphas for all WHOQOL-BREF items in the current study were 0.92 and moderate to good for the four domains (0.56–0.87). Participants rated the WHOQOL-BREF on a 5-point Likert scale; the higher the WHOQOL-BREF mean score, the higher quality of life the participants reported. The WHOQOL-BREF’s total mean score was 3.46 (SD = 0.65). Table 3 presents the remaining descriptive statistics of the four WSS-37 domains.

The IIQ total score significantly correlated with the WHOQOL total score (*r* = 0.56, *p* < 0.05). Continued tests revealed that higher levels of engulfment feelings were significantly correlated with lower psychological (*r* = −0.50, *p* < 0.05) and social (*r* = −0.66, *p* < 0.01) quality of life reported. In addition, higher levels of enrichment feelings were significantly correlated with higher psychological (*r* = 0.67, *p* < 0.01), social (*r* = 0.48, *p* < 0.05) and environmental (*r* = 0.54, *p <* 0.05) quality of life reported.

## 4. Discussion

This article describes the results of the illness identity profile of adults with ASD and its correlations with work-related self-efficacy and health-related quality of life. The results provide preliminary support for the internal reliability of the IIQ as representing the four intended illness identity states (i.e., engulfment, rejection, acceptance and enrichment) in adults with ASD. Previous research with the IIQ indicated its good internal reliability among various populations, such as adolescents and young adults with celiac disease [30], type 1 diabetes [24], refractory epilepsy [29] and adults with a chronic illness [34]. Although the sample size in the current research was relatively small, the results suggest that the IIQ can be useful in assessing illness identity in individuals with ASD. Further research should include a larger sample.

The participants scored significantly higher on the acceptance component than on the other IIQ components. These participants were adults with an average of 9 years of exposure to their diagnosis. Sparud-Lundin et al. [40] suggested that a longer duration of living with an illness allows people to accept their illness and its challenges better. However, this study’s analysis of the positive (acceptance and enrichment) and negative (rejection and engulfment) components revealed no significant difference between them. This indicated that the participants’ negative perceptions are substantially present, and the autism diagnosis is potentially an identity threat [25]. This pattern was identical to findings among individuals with chronic diseases or refractory epilepsy [24,34]. The likeness between negative and positive emphasises that it cannot be taken for granted that a positive perception of living with autism is a secondary profit from participation in various intervention programs or develops with age or time since exposure to diagnosis. Hence, it is imperative that occupational therapists pay specific attention to the issue of autism identity in various intervention programs at any age. This research provides an early understanding of the illness identity concept among individuals with ASD, and much more should be learned about developing intervention programs that promote positive illness identity perceptions in this population.

The high correlations between the IIQ components and the WSS-37 indicate a connection between the participants’ negative feelings about living with autism and their reporting lower perceptions of work-related self-efficacy. Specifically, the low self-efficacy perception in work-related social skills was strongly correlated to feelings of engulfment. Hull et al. [41] suggested that adults with autism reported increased anxiety and stress episodes because, in the real world, they often feel they are losing their authentic sense of identity, sense of grounding and security in who they were. Engulfment is an extreme feeling when individuals feel that autism controls their identity and invades all areas of their lives [24]. Liljeholm and Bergholms’ [42] research among young adults with mental health problems also reinforces the important correlation between identity aspects and work, showing that supporting a positive identity in work may increase their health conditions and self-efficacy. Accordingly, promoting self-efficacy in the work environment may potentially decrease feelings of engulfment. Therefore, it may be worthwhile to develop interventions focusing on reducing engulfment feelings and increasing positive autism identity among individuals with ASD because this might promote their integration into the work world.

Higher levels of engulfment feelings correlated significantly with lower reported psychological and social quality of life. Accordingly, higher levels of enrichment feelings correlated significantly with reporting higher psychological, social and environmental quality of life. These results align with McConachie et al.’s [43] findings that autistic people indicated being positive about their autistic identity as a central aspect particularly relevant to their quality of life. Furthermore, considering the diversity of the ASD diagnosis and the increasing attention in the literature on subthreshold forms of autism [44], assessing the personal aspects, such as illness identity, may be beneficial to understand abilities, feelings, and challenges.

## 5. Limitation and Future Research

This study results should be viewed cautiously given the uncontrolled, nonrandomized design and small sample size. Since the employment rates of adults with ASD in the free work world are meager, there was significant difficulty recruiting subjects. In addition, due to the exceptionally subjective research topic, the point of view was examined through self-report on its advantages and disadvantages. Further research of these viewpoints in a larger sample could expand and strengthen those important initial findings.

## 6. Conclusions

The findings of this study and similar findings in other studies emphasise that people with ASD may feel overwhelmed by their autism and experience an identity disruption that influences the various aspects of their lives [45]. The findings should encourage work and social environments to be more sensitive to the needs of adults with ASD. Allied health professionals and educators can assist individuals with ASD in raising their awareness of how they perceive their diagnosis and how it affects their daily lives. This core identity issue should lead to a more positive perception of their living with autism and a strength-based intervention, which is considered an effective intervention method in this population [39,46].

## Figures and Tables

**Table 1:** Descriptive statistics and Cronbach’s alphas of the illness identity questionnaire (IIQ) components and total score.

Component	Minimum	Maximum	*M*	*SD*	α
Rejection	1.00	4.60	2.62	1.12	0.88
Acceptance	2.20	5.00	3.69	0.82	0.67
Engulfment	1.50	5.00	2.66	1.01	0.86
Enrichment	1.14	4.86	2.61	1.15	0.89
Total negative components	1.46	4.62	3.35	0.92	0.89
Total positive components	1.58	4.58	3.05	.89	0.87
IIQ total score	1.76	4.48	3.21	0.77	0.91

**Table 2:** Descriptive statistics and Cronbach’s alphas of the work-related self-efficacy scale (WSS-37) Domains and total score.

Domain	Minimum	Maximum	*M*	*SD*	α
Vocational service access and career planning	37.50	93.33	63.70	16.04	0.82
Job acquisition	20.00	100.00	64.10	23.98	0.92
Work-related social skills	0.00	100.00	60.31	27.65	0.91
General work skills	32.31	96.15	74.23	19.84	0.92
WSS-37 total score	34.05	94.59	66.72	18.73	0.96

**Table 3:** Descriptive statistics and Cronbach’s alphas of the world health organization quality of life assessment (WHOQOL-BREF) domains and total score.

Domain	Minimum	Maximum	*M*	*SD*	α
Physical health	2.71	4.86	3.59	0.56	0.56
Psychological health	1.50	4.50	3.26	0.80	0.78
Social relationships	1.00	5.00	2.84	1.16	0.87
Environmental	2.50	4.75	3.68	0.70	0.76
WHOQOL-BREF total score	2.19	4.58	3.46	0.65	0.92

## Data Availability

The data presented in this study are available on request from the corresponding author. The data are not publicly available due to ethical restrictions.
